# Auditory Profiles of Classical, Jazz, and Rock Musicians: Genre-Specific Sensitivity to Musical Sound Features

**DOI:** 10.3389/fpsyg.2015.01900

**Published:** 2016-01-07

**Authors:** Mari Tervaniemi, Lauri Janhunen, Stefanie Kruck, Vesa Putkinen, Minna Huotilainen

**Affiliations:** ^1^Cognitive Brain Research Unit, Institute of Behavioural Sciences, University of HelsinkiHelsinki, Finland; ^2^CICERO Learning, University of HelsinkiHelsinki, Finland; ^3^Department of Music, University of JyväskyläJyväskylä, Finland; ^4^Finnish Institute of Occupational HealthHelsinki, Finland

**Keywords:** musical expertise, auditory event-related potentials (ERP), mismatch negativity (MMN), P3a, learning, memory, involuntary attention

## Abstract

When compared with individuals without explicit training in music, adult musicians have facilitated neural functions in several modalities. They also display structural changes in various brain areas, these changes corresponding to the intensity and duration of their musical training. Previous studies have focused on investigating musicians with training in Western classical music. However, musicians involved in different musical genres may display highly differentiated auditory profiles according to the demands set by their genre, i.e., varying importance of different musical sound features. This hypothesis was tested in a novel melody paradigm including deviants in tuning, timbre, rhythm, melody transpositions, and melody contour. Using this paradigm while the participants were watching a silent video and instructed to ignore the sounds, we compared classical, jazz, and rock musicians' and non-musicians' accuracy of neural encoding of the melody. In all groups of participants, all deviants elicited an MMN response, which is a cortical index of deviance discrimination. The strength of the MMN and the subsequent attentional P3a responses reflected the importance of various sound features in each music genre: these automatic brain responses were selectively enhanced to deviants in tuning (classical musicians), timing (classical and jazz musicians), transposition (jazz musicians), and melody contour (jazz and rock musicians). Taken together, these results indicate that musicians with different training history have highly specialized cortical reactivity to sounds which violate the neural template for melody content.

## Introduction

Musical expertise is reflected in the neural architecture of musicians. This is evidenced by both functional (for reviews, see Münte et al., 2002; Jäncke, 2009; Pantev and Herholz, 2011) and structural (e.g., Schlaug et al., 1995; Gaser and Schlaug, 2003; Bengtsson et al., 2005; Steele et al., 2013) findings in adult musicians as well as in children who received music training (Hyde et al., 2009; Putkinen et al., 2014).

In particular, electrical auditory brain potentials reflecting the successive stages of sound processing are either stronger and/or earlier in musicians than in non-musicians. These cortical reactions include P1 (Schneider et al., 2002), N1 (Pantev et al., 1998, 2001), and P2 (Shahin et al., 2003; Seppänen et al., 2012a), which are evoked by regular sounds. They also include mismatch negativity (MMN) as well as P3a, which are evoked by irregular “deviant” sounds among regular sequences consisting of music sounds (see, e.g., Koelsch et al., 1999; Rüsseler et al., 2001; Fujioka et al., 2004, 2005; Virtala et al., 2014; P3a, see, e.g., Seppänen et al., 2012b) or speech sounds in adults (Kühnis et al., 2013) and in children (Chobert et al., 2011). MMN and P3a responses signal the neurocognitive discrepancy between the expected and the encountered sound information (MMN: Kujala et al., 2007; P3a: Escera et al., 2000).

In these pioneering studies in the neurosciences of music, musical expertise was conceptualized to originate from expertise in performing classical Western music. However, even at the highest levels of music education, other musical traditions, and genres are now appreciated and taught. Thus, the concept of musical expertise needs to cover other musical genres as well. Moreover, due to their high motivation and intense involvement in musical activities, even participants without professional status or formal training in music but with an identity as a musician need to be taken into account when investigating the neurocognitive determinants of musical expertise. Such individuals earn their living from other professions but spend their free time (and even financial resources) in rehearsing and performing music.

Since musical genres differ from each other in various acoustical and musical features, it is hypothesized that musicians who are active predominantly in one genre have a different auditory sound encoding profile when compared with other musicians or non-musicians (Tervaniemi, 2009, 2012). Indeed, in two pioneering studies on jazz musicians (Vuust et al., 2005) and rock musicians (Tervaniemi et al., 2006), this was tentatively shown when comparing these musicians with non-musicians. Additionally, musicians who usually play without sheet music were found to have facilitated MMN responses to contour changes in melodic patterns when compared with musicians who train and perform by using musical notation (Tervaniemi et al., 2001; Seppänen et al., 2007).

Recently an auditory event-related potential (ERP) study used a novel paradigm which allows one to systematically determine the degree of the discrepancy between the expected (standard) and unexpected (deviant) sounds for several sound features in an Alberti bass setting (Vuust et al., 2011, 2012). It was hypothesized that the more frequently a given group of musicians encounters any of the deviant sound changes, the more pronounced is their neural reaction to that change. The auditory ERPs were investigated in rock, classical, and jazz musicians and also in a group of non-musicians. Participants were presented with a sound sequence consisting of regular pattern of sounds which included six acoustic changes relevant for musical processing in different musical genres. Specifically, five of the six musical features were aspects of musical sound that have previously been shown to elicit larger MMNs in musicians than in non-musicians: pitch mistuning, timbre, sound-source location, intensity, and rhythm. A pitch slide, which is common in improvisational music, particularly jazz, was also included. The MMN evidence indicated that jazz musicians had larger MMN amplitudes than all other groups of participants across the six different sound features, indicating a greater overall sensitivity of jazz musicians to auditory changes of any kind. This was also reflected in the AMMA musicality test, reported in the same paper: jazz musicians scored higher in the Tonal subtest when compared with rock musicians and non-musicians. In the Rhythm subtest, scores were worst in non-musicians while jazz musicians scored higher than rock musicians.

Here we expected to find a more fine-grained pattern of musicians' auditory sensitivity to different sound features by using a new melodic MMN paradigm (Putkinen et al., 2014; Tervaniemi et al., 2014) in which a melody of about 2 s is presented in a loop. As introduced below, it has several kinds of deviant sounds in terms of pitch, timbre, harmony, and timing. Importantly, we use both low-level deviants holding the melody content constant and high-level deviants which modify the successive melody presentations. Our hypotheses were that (1) musicians would display larger MMN responses than non-musicians, implying more accurate auditory memory traces in musicians (2) musicians would differ from each other in MMN amplitude, with larger MMN responses reflecting the importance of a given sound feature in their genre (classical: pitch, jazz: transpositions).

## Methods

### Participants

In total, there were 60 healthy adult participants involved in EEG recordings. Due to noisy EEG, six participants were excluded from the final analyses. The remaining 54 participants were divided into four groups according to their musical background.

#### Rock musicians

This group included 19 subjects, from which 13 were males and 6 females (average age 28 years, SD 6 years). They had started to play at the age of 14 on average (SD 10 years). They were currently involved in band activities about 7 h a week (see Table 1).

**Table 1:** **Background information of musicians**.

	**Starting age**	**Years played**	**Main instrument**	**Gender**
**Rock**					
	9	18	Accordion	m
	47	3	Accordion	m
	24	7	Bass	m
	17	12	Drums	m
	15	13	Guitar	m
	13	15	Guitar	m
	15	13	Guitar	f
	15	12	Guitar	m
	10	17	Guitar	m
	7	19	Guitar	f
	12	15	Guitar/Sing	m
	12	16	Guitar/Sing	m
	21	6	Keyboards	m
	6	21	Piano	f
	7	23	Piano/keyboards	m
	8	20	Sing	m
	5	18	Sing	f
	12	9	Steelpan	f
	6	14	Violin	f
*M*	14	14		
**Classical**					
	5	18	Viola	m
	7	19	Cello	m
	9	42	Clarinet	m
	16	30	Double bass	m
	3	31	Piano	f
	9	18	Piano	f
	5	40	Piano	m
	6	19	Piano	f
	6	20	Piano	f
	9	32	Sing/compose	m
	10	18	Violin	f
	14	27	Violin	f
*M*	8	26		
**Jazz**					
	10	31	Clarinet	m
	16	13	Double bass	m
	10	15	Drums	m
	10	16	Drums	m
	10	15	Electric bass	m
	17	14	Guitar	m
	10	34	Guitar	m
	16	26	Guitar	m
	7	27	Piano	f
	9	14	Saxophone	f
	13	30	Trumpet	m
*M*	12	21		

#### Classical musicians

Classical musicians consisted of 12 subjects, from which 6 were males and 6 females (their average age was 34 years, SD 10 years). They had started to play at the age of 8 years on average (SD 4 years). They were currently practicing or performing music about 20 h a week.

#### Jazz musicians

Jazz musicians included 11 subjects, from which 9 were males and 2 females (average age 33 years, SD 8 years). They had started to play at the age of 12 (SD 3 years). They were currently practicing or performing music about 20 h a week.

Additionally, a group of *Non-musicians* included 12 subjects, from which 8 were males and 5 females (average age 27 years, SD 2 years). They had no formal music training apart from music lessons at the primary and secondary school, and were never taught to play an instrument except for one subject who had played the piano for less than a year when he was 8 years old.

### Stimuli

The stimuli, used also by Putkinen et al. (2014) and Tervaniemi et al. (2014) were as follows. Digital piano tones were used to create a short melody pattern that was in accordance with Western tonal rules and was recursively repeated. Short melodies always started with a triad (300 ms) which was followed by four tones and an ending tone. There was a 50 ms gap between successive tones andhe ending tone was 575 ms in duration. There was also a 125 ms gap between each melody. Therefore, one melody lasted for 2100 ms.

**Figure 1:**
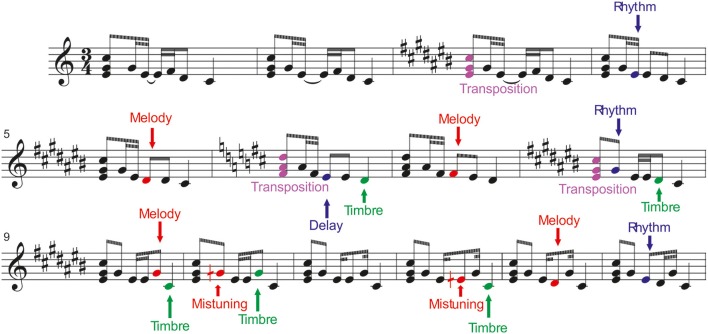
**Stimulation paradigm**. The melodies were presented via headphones while the participants were watching a silenced movie. (From Tervaniemi et al., 2014, with permission).

Six different deviant tones were included in the melodies. They were divided into low-level changes, which did not change the melody, and into high-level changes which altered the melodic contour. For illustration, see Figure 1.

#### Low-level changes

1. *Mistuning* (half of a semitone, 3% of the fundamental frequency of the sound) could occur in the first, second, or fourth tone of the melody.
2. *Timbre deviant* (flute instead of a piano) could occur in the first, third, or fourth tone of the melodies, or in the ending tone.
3. *Timing delay* (= 100 ms silent gap) could occur in the first, second, or third tone in 8% of the melodies, or in the end of a melody.

#### High-level changes

1. *Melody modulation* was presented as a pitch change of the third or fourth tone of the melody. It slightly changed the prevailing melody and continued until a new melody modulation was introduced.
2. *Rhythm modulation* (= reversal of the duration of two sequential tones) could occur in the second or third tone.
3. *Transposition* (one semitone up or down) occurred in the first triad. After chord transposition, the following melodies retained the converted key until a new chord transposition was introduced.

### Procedure

During the EEG recordings, subjects were sitting in a dimly lit EEG chamber in a comfortable chair. They were instructed to watch a silent nature documentary with subtitles while stimuli were presented via headphones. The EEG recording was preceded by a 10-min session during which the participants were asked to listen to three self-selected music samples while their EEG and physiological responses were recorded. These data will be reported elsewhere.

The experiment was approved by the Ethical committee of the former Department of Psychology, University of Helsinki. The participants were rewarded with movie tickets for their participation.

#### EEG recordings

The recordings were conducted in an acoustically and electromagnetically shielded room (Euroshield Ltd., Finland) of the Institute of Behavioural Sciences, University of Helsinki.

The EEG was recorded with the BioSemi system with a sampling rate of 4096 Hz and a 64-electrode EEG-cap with six additional silver-chloride (Ag/AgCl) electrodes. They were attached on the mastoids, on the tip of the nose, under the right eye (for EOG monitoring) and two on EMG-related (electromyography) sites on the left cheek and over the left eyebrow. The average of mastoid electrodes was used as a reference during the offline analyses. The EOG electrode was used to extract eyeblink artifacts.

Hearing thresholds were individually obtained by averaging five tests of just-audible sounds. The volume level was set as 60 dB above this threshold.

### Data analysis

The data were analyzed using EEGLab 6.01. They were filtered with a high-pass cut-off of 1 Hz (due to fast stimulation rate) and a low-pass cut-off of 45 Hz. Though the high-pass of 1 Hz might not be optimal for P3a, we justify this choice by the fact that identical filter settings could be used for both MMN and P3a responses. The EEG was divided into epochs from −100 to 600 ms (baseline −100 ms before sound onset at 0 ms). Extreme amplitude values were manually rejected from the data. Channels of poor quality were interpolated manually.

Independent Component Analysis (ICA) was conducted for all participants' data to further ensure that artifacts due to an eye blink or to a channel with poor contact were excluded and a rejection threshold of ±100 μV was applied. For all participants included in the analyses, at least 80% of the trials were accepted.

Finally, manual rejection removed epochs that still displayed clear EOG-fluctuations or otherwise abnormally massive waveforms related to muscle activation, shown as a hundred to a thousand times greater magnitude than the cortical signals of interest.

Amplitudes were quantified from individual difference waves using a 40 ms time window centered on the peak of the MMN and P3a components in the grand-average difference waves. Mean values from these time windows were calculated for F3, Fz, F4, C3, Cz, C4, P3, Pz, and P4.

### Statistical analysis

The group differences in the MMN amplitudes were tested first with an 4 × 3 × 3 × 9 mixed model omnibus ANOVA with Group (Rock Musicians/Classical Musicians/Jazz Musicians/Non-musicians) as a between subject factor and Left-Right (Left/Middle/Right), Anterior-Posterior (Anterior/Central/Posterior) and Deviant (Mistuning, Timbre middle, Timbre end, Timing delay middle, Timing delay end, Melody modulation, Rhythmic modulation short, Rhythmic modulation long, Transposition) as within-subject factors. This omnibus ANOVA was followed by separate 4 × 3 × 3 mixed-model ANOVAs for each deviant type with the factors Group, Left-Right, and Anterior-Posterior. Since the P3a was not elicited by all deviants or was elicited only in the musician groups, an omnibus ANOVA similar to the one described above could not be performed for the P3a. Instead, separate ANOVAs were conducted for those deviants that elicited the P3a with the factors Group (either with all four groups or the musician groups only), Left-Right and Anterior-Posterior. Bonferroni correction was used for the *post-hoc* pairwise comparisons.

In analysing the topography, left electrodes were F3, C3, P3, middle electrodes were Fz, Cz, Pz, and right electrodes were F4, C4, P4. Anterior electrodes were formed by F-line, central electrodes by C-line, and posterior electrodes by P-line.

## Results

**Figure 2:**
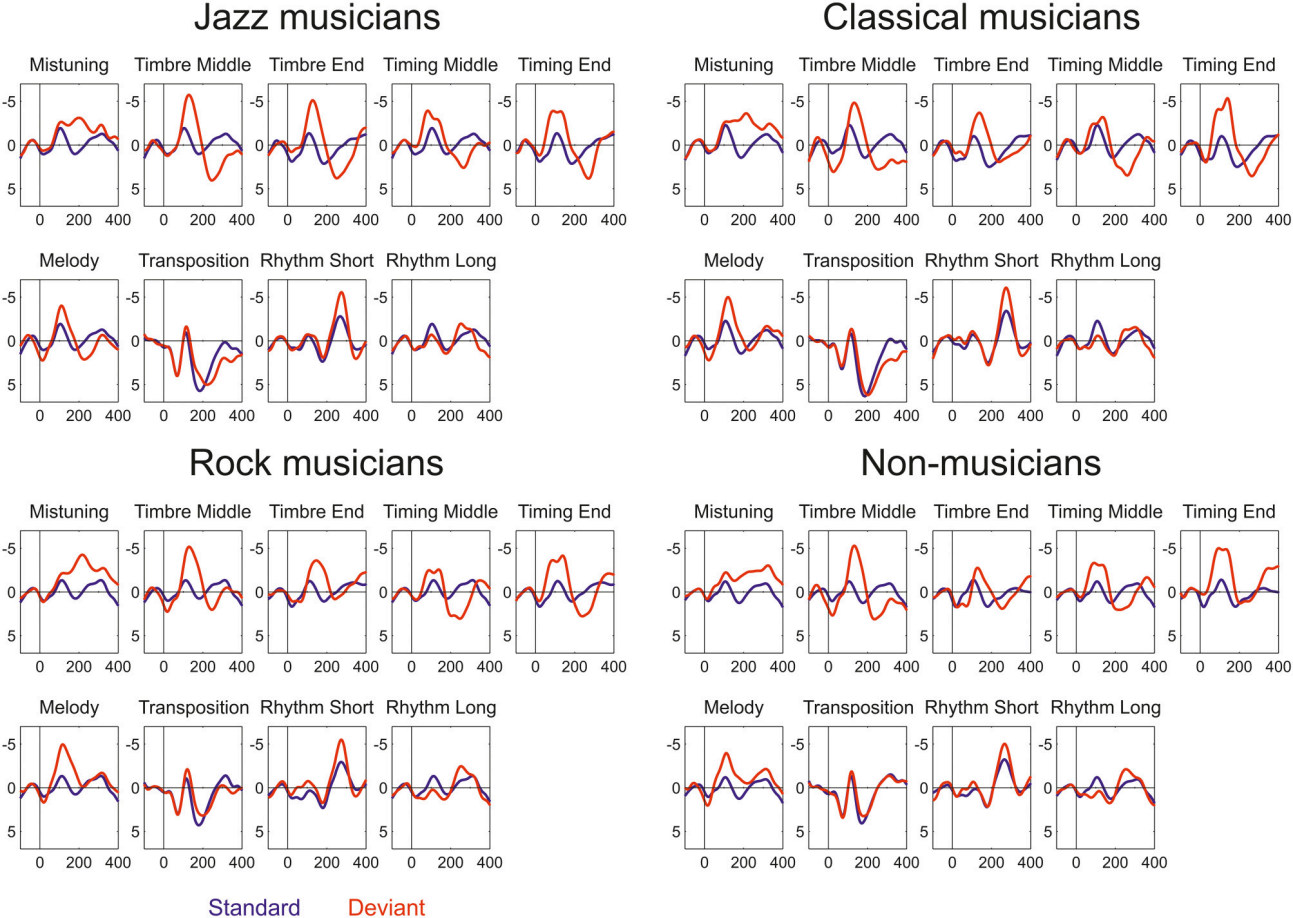
**Event-related potentials elicited by standard (blue) and deviant (red) sounds within the melodies in Jazz musicians, Classical musicians, Rock musicians, and Non-musicians**.

All deviant sounds elicited MMN responses in all participant groups (Figure 2). Omnibus ANOVA indicated that there is significant Anterior-posterior^*^Group interaction (Interaction effect: *F*_(5.63, 95.73)_ = 4.5, *p* < 0.001) and marginally significant interaction between Group, Deviant, and Anterior-Posterior dimension (Interaction effect: *F*_(21.41, 364.04)_ = 1.5, *p* = 0.092). Occasionally, the MMN was followed by a P3a.

The following are results first for the low-level deviants (mistuning, timbre, and timing delay) and then for the high-level deviants (key, melody modulation, rhythm modulation) whose occurrence changed the melodic structure.

### Low-level deviants

#### Mistuning

The mistuning MMN was frontally maximal [Main effect: Anterior-posterior (*F*_(1.38, 70.18)_ = 123.1, *P* < 0.001)] with topography that differed between the participant groups [Interaction effect: Anterior-posterior × Group (*F*_(4.13, 70.18)_ = 4.39, *p* < 0.01); see Table 2]. *Post-hoc* tests indicated that the MMN was anteriorly larger in classical musicians when compared to non-musicians (*p* < 0.05).

**Table 2:** **ANOVA results in which Group factor provides significant Main or Interaction results**.

	**Anterior-Posterior** × **Group**			**Left-Right** × **Group**			**Anterior-Posterior** × **Left-Right** × **Group**			**Group**		
	**df**	***F***	***p***	**df**	***F***	**p**	**df**	***F***	***p***	**df**	***F***	***p***
Mistuning MMN	4.13, 70.17	4.39	**0.003**	5.53, 94.07	0.76	0.590	8.16, 138.77	1.223	0.290	3.51	2.08	0.115
Timbre (within) MMN	4.66, 79.29	1.27	0.288	5.98, 101.58	1.44	0.205	6.33, 107.58	1.871	0.089	3.51	0.90	0.447
Timbre (within) P3a	4.05, 68.91	0.67	0.616	5.47, 92.93	0.24	0.955	8.51, 144.72	1.289	0.251	3.51	1.60	0.201
Timbre (end) MMN	4.27, 72.51	3.32	**0.013**	5.86, 99.62	0.91	0.488	10.36, 176.17	1.208	0.287	3.51	0.73	0.540
Timbre (end) P3a	5.61, 95.33	1.29	0.271	5.51, 93.69	0.50	0.789	7.88, 133.91	1.002	0.437	3.51	3.03	**0.037**
Timing (within)MMN	4.62, 78.61	1.18	0.326	4.65, 79.03	1.22	0.308	6.50, 110.49	1.153	0.337	3.51	1.25	0.300
Timing (within) P3a	4.14, 70.44	1.37	0.252	4.94, 83.93	0.28	0.923	9.15, 155.60	1.777	0.075	3.51	0.71	0.549
Timing (end) MMN	4.48, 76.10	1.81	0.129	4.75, 80.70	0.77	0.565	6.47, 110.06	0.891	0.510	3.51	0.58	0.633
Timing (end) P3a	4.17, 70.94	0.16	0.962	4.19, 71.20	0.47	0.767	6.09, 103.49	0.992	0.435	3.51	5.19	**0.003**
Melody MMN	4.65, 79.09	1.74	0.141	4.73, 80.44	0.78	0.563	6.61, 112.34	1.043	0.404	3.51	2.50	0.070
Melody P3a	2.61, 50.85	1.38	0.261	3.60, 70.12	0.68	0.592	4.38, 85.43	0.351	0.859	2.39	5.37	**0.009**
Transposition MMN	4.75, 80.74	1.39	0.238	5.46, 92.89	2.78	**0.019**	9.46, 160.78	1.477	0.157	3.51	2.57	0.065
Transposition P3a	2.68, 52.23	0.90	0.437	3.51, 68.47	0.90	0.459	4.74, 92.43	0.048	0.998	2.39	5.37	**0.009**
Rhythm (short) MMN	4.78, 81.29	2.29	0.056	4.74, 80.57	0.46	0.797	5.39, 91.64	0.692	0.641	3.51	0.07	0.976
Rhythm (long) MMN	4.46, 75.77	1.19	0.324	5.48, 93.20	1.02	0.415	7.26, 123.37	2.131	**0.043**	3.51	0.37	0.773

*Statistically significant values are with bold*.

#### Timbre (within a melody)

##### MMN

The timbre-MMN was frontally maximal [Main effect: Anterior-posterior (*F*_(1.42, 72.52)_ = 75.1, *P* < 0.001)]. No statistically significant amplitude differences between the groups were found.

##### P3a

MMN was followed by the P3a which had a fronto-central maximum [Interaction effect: Left-Right × Anterior-posterior (*F*_(8.51, 144.72)_ = 6076, *p* < 0.01)]. The P3a amplitude did not differ between the groups.

#### Timbre (melody ending)

##### MMN

The amplitude of the frontally maximal MMN [Main effect: Anterior-posterior (*F*_(1.42, 72.52)_ = 75.08, *p* < 0.001)] differed between the groups along the anterior-posterior axis [Anterior-posterior × Group (*F*_(4.27, 72.52)_ = 3.32, *p* < 0.05)]. However, *post-hoc* tests did not yield any significant differences between the groups.

##### P3a

The P3a had a fronto-central maximum [Interaction effect: Anterior-posterior × Left-right (*F*_(2.63, 133.91)_ = 3.97, *p* < 0.05)]. In addition, its amplitude differed between the groups [Main effect: Group (*F*_(3.51)_ = 3.03, *p* < 0.05)]. The P3a was significantly larger in Jazz musicians than in Rock musicians (*p* < 0.05).

#### Timing delay (within the melody)

##### MMN

Timing delays elicited a frontally maximal MMN [Interaction effect: Anterior-posterior (*F*_(1.54, 78.61)_ = 62.49, *p* < 0.001)] without differences in topography or amplitude between the groups.

##### P3a

The P3a was largest at the fronto-central electrodes [Interaction effect: Anterior-posterior × Left-right (*F*_(3.05, 155.6)_ = 6.52, *p* < 0.001)]. There were no differences between groups.

#### Timing delay (end of the melody)

##### MMN

Timing delays elicited an MMN that was maximal over the frontal electrodes [Main effect: Anterior-posterior (*F*_(1.49, 76.1)_ = 70.43, *p* < 0.001)]. There were no differences in MMN amplitude or topography between the groups.

##### P3a

P3a was largest frontally and at the midline electrodes [Main effects: Anterior-posterior (*F*_(1.39, 70.1)_ = 12.32, *p* < 0.001; Left-right *F*_(1.4, 71.2)_ = 8.12, *p* < 0.01)]. In addition, its amplitude differed between the groups [Main effect: Group (*F*_(3, 5)_ = 5.19, *p* < 0.01)], i.e., the P3a was larger in classical (*p* < 0.01) and jazz musicians (*p* < 0.05) when compared to non-musicians.

### High-level deviants

#### Melody modulation

Melody modulations elicited MMN in all participant groups, which were followed by the P3a in all other groups except non-musicians.

##### MMN

The Melody MMN was largest over the frontal and central electrodes [Main effects: Anterior-posterior (*F*_(1.55, 79.09)_ = 90.28, *p* < 0.001; Left-right *F*_(1.58, 80.44)_ = 3.67, *p* < 0.05)]. MMN amplitude differed marginally between the participant groups [Main effect: Group (*F*_(3, 51)_ = 2.5, *p* = 0.07)]. *Post-hoc* tests indicated that the MMN amplitude was marginally larger in rock musicians when compared to jazz musicians (*p* = 0.053).

##### P3a

The P3a had a frontal maximum [Main effect: Anterior-posterior (*F*_(1.3, 50.85)_ = 5.7, *p* < 0.05)]. Additionally, there was a group main effect of P3a amplitude [*F*_(2, 39)_ = 5.37, *p* < 0.01], which *post-hoc* tests indicated was larger in jazz musicians than in classical and rock musicians (*p* < 0.05).

#### Transposition

##### MMN

The transposition MMN had a fronto-central maximum [Interaction effect: Anterior-posterior × Left-right (*F*_(3.15, 160.78)_ = 4.25, *p* < 0.01)]. There was a significant difference in MMN topography along the left-right axis [Interaction effect: Left-right × Group (*F*_(5.46, 92.89)_ = 2.78, *p* < 0.019)]. *Post-hoc* tests indicated that the MMN amplitude was larger in jazz and rock musicians than in non-musicians above the right hemisphere (*p* < 0.05).

##### P3a

The P3a was largest frontally [Main effect: Anterior-posterior (*F*_(1.34, 52.23)_ = 24.07, *p* < 0.001)] and its amplitude differed between the groups [Main effect: Group (*F*_(2, 39)_ = 5.37, *p* < 0.01)]. *Post-hoc* tests indicated that the P3a was larger in jazz musicians than in rock musicians (*p* < 0.05) and marginally larger in classical musicians than in rock musicians (*p* = 0.05).

#### Rhythm modulation (short tone within the melody)

##### MMN

The MMN was maximal over the frontal and midline electrodes [Main effects: Anterior-posterior (*F*_(1.59, 81.29)_ = 20.54, *p* < 0.001; Left-right *F*_(1.58, 80.57)_ = 4.7, *p* < 0.05)]. The MMN topography differed marginally between the groups [Interaction effect: Anterior-posterior × Group (*F*_(4.78, 81.29)_ = 2.29, *p* = 0.056)]. However, no significant group differences in MMN topography were found in *post-hoc* analyses.

#### Rhythm modulation (long tone within the melody)

##### MMN

The MMN was frontally maximal [Main effect: Anterior-posterior (*F*_(1.47, 73.44)_ = 12.52, *p* < 0.001)]. The topography of the MMN differed significantly between the groups [Interaction effect: Group × Anterior-posterior × Left-right (*F*_(7.26, 123.37)_ = 2.13, *p* < 0.05)]. However, the *post-hoc* test did not reveal any significant group differences in MMN amplitude at any of the individual electrodes.

## Discussion

We compared the auditory profiles of classical, jazz, and rock musicians to those of non-musicians in encoding various sound features. In contrast to traditional ERP paradigms, we used a novel melodic MMN paradigm in order to approach realistic musical setting. It allows one to determine the extent of discrepancy between the expected (standard) and unexpected (deviant) sounds of low and high levels of complexity. It was hypothesized that the more frequently a given group of musicians encounters any of the sound changes (in terms of deviants), the more pronounced is their neurocognitive MMN response to that change.

First, in the case of the low-level changes, we found out that mistuned sounds among the melody evoked a frontally enhanced MMN especially in classical musicians when compared to non-musicians. We also found that the timing delays in the end of the melody evoked a larger P3a in classical and jazz musicians when compared to non-musicians. In parallel, timbre deviants in the end of the melody evoked larger P3a in jazz musicians when compared to Rock musicians. From the current mistuning MMN findings as well as previous results, we can conclude that musical training in classical Western music is associated with auditory neurocognitive functions which are highly sensitive to mistuned notes within a musical context (cf. Koelsch et al., 1999 who showed that violin players are sensitive to encoding mistuned sounds when they are among chords but not among sinusoidal tones). We suggest that the current MMN findings for group differences in timing delay and timbre, selectively in the end of the melody, reflect the accuracy of expectation for the onset of the last tone of a given melodic sequence, which becomes more accurate with explicit music training in classical or jazz music, genres in which the timing of the last sound has expressive importance.

Second and most importantly, in the case of high-level changes, melody modulation evoked MMN which was larger in rock musicians than in jazz musicians. However, the subsequent P3a was larger in jazz musicians than in classical or rock musicians. This suggests that the most reliable ERP signal here to differentiate the groups of musicians is the P3a, which reflects the involvement of involuntary attention (Escera et al., 2000), or, alternatively, the multi-stage process of sound evaluation which leads to attention shift (Schröger et al., 2014). Although the rock musicians showed enhanced MMN to melody modulation when compared to jazz musicians, this result can be explained by the rapid onset of the subsequent P3a in jazz musicians which might have been already active during the original MMN response. This may indicate that attention may be more readily shifted in jazz musicians, particularly in the context of melody modulations. Correspondingly, in melodies with high-level changes, melody transpositions evoked more pronounced MMN in jazz and rock musicians than in non-musicians. However, the subsequent P3a was larger in jazz and classical musicians than in rock musicians. This also suggests that here, the most reliable ERP index to differentiate the groups of musicians is the P3a.

**Figure 3:**
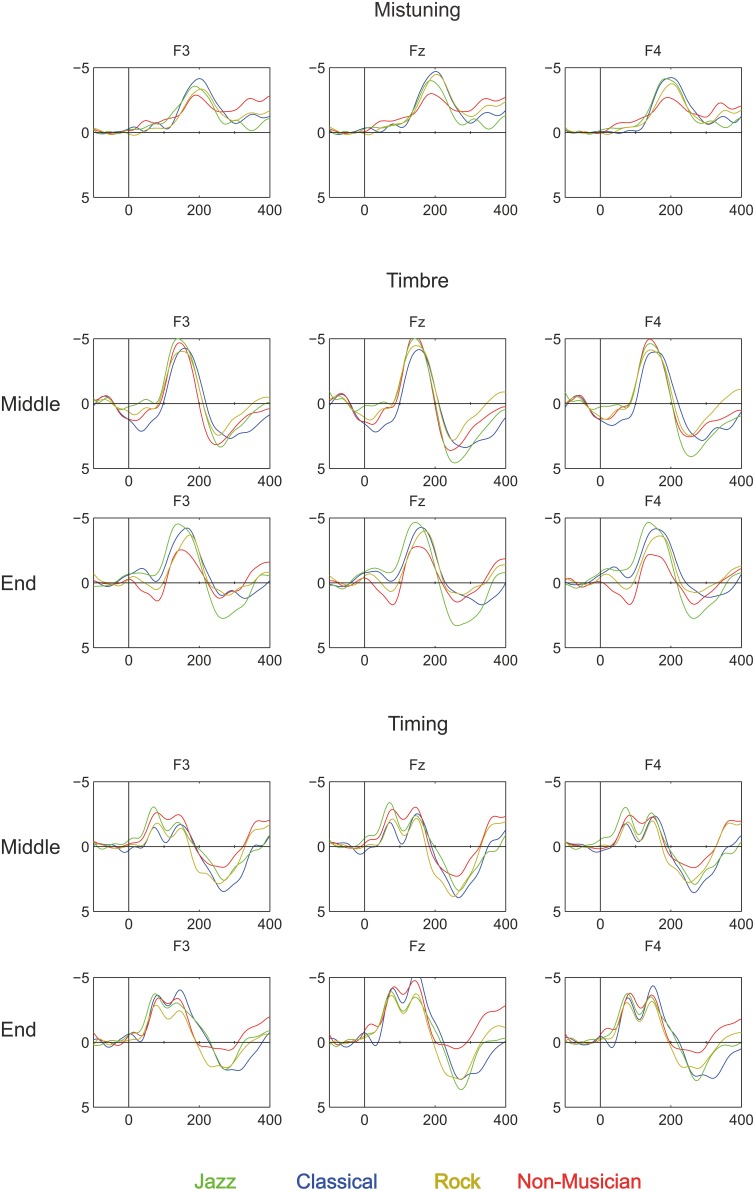
**Brain responses (ERP to deviant melody subtracted from the ERP evoked by the standard melody) in Classical musicians (blue line), Jazz musicians (green line), Rock musicians (yellow line), and Non-musicians (red line) to Mistuning, Timbre, and Timing delay (Rhythm mistake)**. These deviants were introduced in the melody but they did not modulate the continuation of the melody.

**Figure 4:**
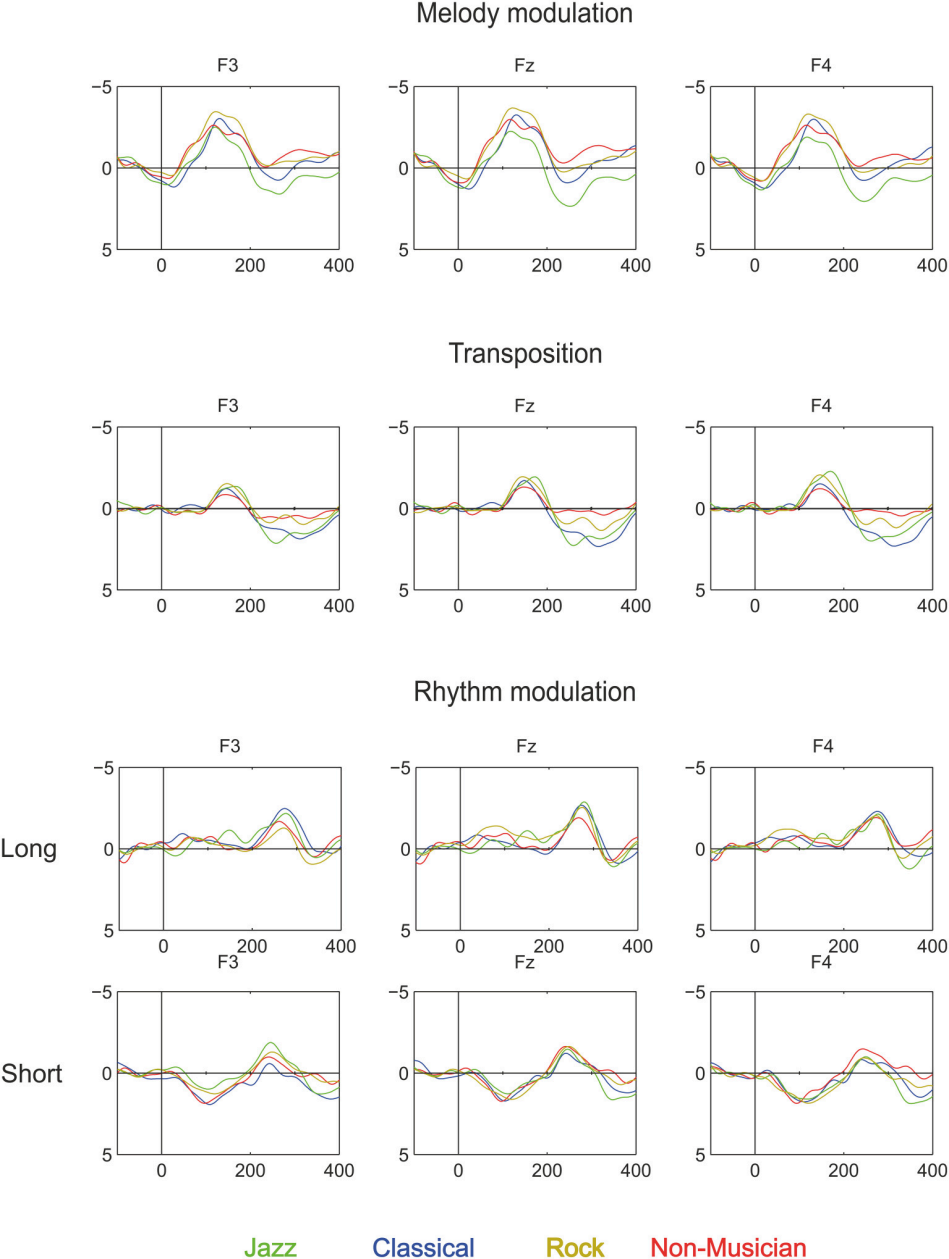
**Brain responses to Rhythm modulation, Melody modulation, and Transposition in Classical musicians (blue line), Jazz musicians (green line), Rock musicians (yellow line), and Non-musicians (red line)**. These deviants were introduced in the melody and they modulated its continuation.

Third, our results show that in all participant groups, deviant sounds evoked an MMN, see Figures 3, 4. Taking into account the complexity of the sound material and the instruction to watch the movie instead of listening to the sounds, the present data provide unique evidence about implicit knowledge of the regularities of the Western musical system which is encoded by all individuals of a given musical culture, even when they lack explicit training in music (as in the case of non-musicians). Thus, this finding replicates and extends our recent findings obtained by using the same melodic paradigm to compare folk musicians and non-musicians (Tervaniemi et al., 2014). To our knowledge, only in Brattico et al. (2006) were melodies with constantly varying contours used to successfully probe the existence of the pitch-related long-term memory templates in musically non-trained participants. The present stimulation paradigm thus offer a valuable extension to the literature by showing that the musically non-trained participants are able to preattentively encode both low- and high-level changes in the melodic contour. Interestingly, this ability appears to emerge relatively slowly during development in individuals without musical training. A previous study showed that MMN responses obtained with the current paradigm in children without musical training were clearly smaller than those of musically trained children and did not show evidence of age-related increase during school-age development (Putkinen et al., 2014; see also below).

As an interim conclusion, the present data confirm our hypothesis that adult musicians display different sound-related MMN and P3a responses as a function of their genre of musical training and can be said to have differential auditory profiles. It is also important to note that first, the P3a might be a more sensitive index of music familiarity, and second, that even adult participants without explicit training in music displayed significant MMNs, indicating that their auditory system could encode the relatively complex melody preattentively.

A main limitation of the current study is that the groups of musicians were not matched according to several background variables, e.g., gender and age of onset of music training. To avoid misinterpretations of the results based on this unbalance, it will be necessary in the future to pay specific attention to match the groups of musicians as carefully as possible in terms of gender, age of onset of musical practice, and the formal training they received. However, this is quite a challenge since the genres and their musicians unanimously differ in these respects and as a result have different profiles not only in their auditory processes but also in many social and training aspects. So, by matching such background variables we might actually lose the phenomenon under interest—the great variety in musicians.

Another limitation of the current approach is that when investigating the neurocognitive abilities in adults only, we are not able to collect behavioral or neurophysiological data prior to the onset of their training (for a recent discussion, see Barrett et al., 2013). Because of this, we cannot reliably conclude whether there were some differences in neurocognitive function between the current groups of individuals prior to their involvement (or non-involvement) in musical training. It might be that such individual differences both in perceptual skills and in motivational factors were already present and influenced the decisions made by children and their parents when there were several hobbies available.

Even so, already in 2009, Hyde et al. reported a pioneering study about the effects of musical training at the structural level of the brain in children between 4 and 6 years of age (Hyde et al., 2009). At the onset of training, there were no structural differences between the groups. However, individual training of only 15 months in keyboard playing (compared with group-based lessons in drumming) modified the brain structure at the auditory and motor areas as well as in the corpus callosum. If we take into account that the control children also had training in music, it is highly interesting to note that individually-driven and thus more intense music training made a more profound effect on the brain structure even in such a short time frame when compared with a group-based training.

Even more relevant findings in the current context were recently obtained by Putkinen et al. (2014) who, using the same melodic MMN paradigm as in the current paper, made a longitudinal analysis of the development of MMN in 9–13 year old children learning to play an instrument according to classical Western tradition. When compared to the control children without music-related hobbies, the musically trained children displayed enlarged MMNs for melody modulations by age 13 and for rhythm modulations, timbre deviants, and mistuned tones at the age of 11. In addition, the MMN elicited by rhythm deviants was larger in amplitude in the musically trained than in the non-trained children at age 13. Importantly, since no group differences were found at age 9, the later enhancement of the MMN in the musically trained children resulted from training and not pre-existing difference between the groups. It is also important to note that on the basis of these findings, the development of sensitivity to different sound features is not uniform but differs between the features.

In sum, the current findings suggest that long-term training of musicians in a given musical genre can modulate their auditory processes as indicated by MMN and P3a. They also suggest that the neural generators differ between the groups of musicians. To elucidate this further, an ongoing project using combined EEG and MEG recordings will find out the functional specialization of the auditory encoding in different musicians (Kliuchko et al., in preparation). Importantly, this project has in its use both Alberti bass –based paradigm developed by Vuust et al. (2011, 2012) and the current melody-based paradigm (Putkinen et al., 2014; Tervaniemi et al., 2014) enabling direct comparisons between the paradigms.

### Conflict of interest statement

The authors declare that the research was conducted in the absence of any commercial or financial relationships that could be construed as a potential conflict of interest. The reviewer Sammler and handling Editor Kotz declared their shared affiliation, and the handling Editor states that the process nevertheless met the standards of a fair and objective review.

## References

[B1] Barrett K. C. Ashley R. Strait D. L. Kraus N. (2013). Art and science: how musical training shapes the brain. Front. Psychol. 4:713. 10.3389/fpsyg.2013.00713 24137142 PMC3797461

[B2] Bengtsson S. L. Nagy Z. Skare S. Forsman L. Forssberg H. Ullén F. (2005). Extensive piano practicing has regionally specific effects on white matter development. Nat. Neurosci. 8, 1148–1150. 10.1038/nn1516 16116456

[B3] Brattico E. Tervaniemi M. Näätänen R. Peretz I. (2006). Musical scale properties are automatically processed in the human auditory cortex. Brain Res. 1117, 162–174. 10.1016/j.brainres.2006.08.023 16963000

[B4] Chobert J. Marie C. François C. Schön D. Besson M. (2011). Enhanced passive and active processing of syllables in musician children. J. Cogn. Neurosci. 23, 3874–3487. 10.1162/jocn_a_00088 21736456

[B5] Escera C. Alho K. Schröger E. Winkler I. (2000). Involuntary attention and distractibility as evaluated with event-related potentials. Audiol. Neurootol. 5, 151–166. 10.1159/000013877 10859410

[B6] Fujioka T. Trainor L. J. Ross B. Kakigi R. Pantev C. (2005). Automatic encoding of polyphonic melodies in musicians and nonmusicians. J. Cogn. Neurosci. 17, 1578–1592. 10.1162/089892905774597263 16269098

[B7] Fujioka T. Trainor L. Ross B. Kakigi R. Pantev C. (2004). Musical training enhances automatic encoding of melodic contour and interval structure. J. Cogn. Neurosci. 16, 1010–1021. 10.1162/0898929041502706 15298788

[B8] Gaser C. Schlaug G. (2003). Brain structures differ between musicians and non-musicians. J. Neurosci. 23, 9240–9245. 10.1016/S1053-8119(01)92488-7 14534258 PMC6740845

[B9] Hyde K. L. Lerch J. Norton A. Forgeard M. Winner E. Evans A. C.. (2009). Musical training shapes structural brain development. J. Neurosci. 29, 3019–3025. 10.1523/JNEUROSCI.5118-08.2009 19279238 PMC2996392

[B10] Jäncke L. (2009). The plastic human brain. Restor. Neurol. Neurosci. 27, 521–538. 10.3233/RNN-2009-0519 19847074

[B11] Koelsch S. Schröger E. Tervaniemi M. (1999). Superior attentive and pre-attentive auditory processing in musicians. Neuroreport 10, 1309–1313. 10.1097/00001756-199904260-00029 10363945

[B12] Kühnis J. Elmer S. Meyer M. Jäncke L. (2013). The encoding of vowels and temporal speech cues in the auditory cortex of professional musicians: an EEG study. Neuropsychologia 51, 1608–1618. 10.1016/j.neuropsychologia.2013.04.007 23664833

[B13] Kujala T. Tervaniemi M. Schröger E. (2007). The mismatch negativity in cognitive and clinical neuroscience: theoretical and methodological considerations. Biol. Psychol. 74, 1–19. 10.1016/j.biopsycho.2006.06.001 16844278

[B14] Münte T. F. Altenmüller E. Jäncke L. (2002). The musician's brain as a model of neuroplasticity. Nat. Rev. Neurosci. 3, 473–478. 12042882 10.1038/nrn843

[B15] Pantev C. Herholz S. C. (2011). Plasticity of the human auditory cortex related to musical training. Neurosci. Biobehav. Rev. 35, 2140–2154. 10.1016/j.neubiorev.2011.06.010 21763342

[B16] Pantev C. Oostenveld R. Engelien A. Ross B. Roberts L. E. Hoke M. (1998). Increased auditory cortical representation in musicians. Nature 392, 811–814. 10.1038/33918 9572139

[B17] Pantev C. Roberts L. E. Schulz M. Engelien A. Ross B. (2001). Timbre-specific enhancement of auditory cortical representations in musicians. Neuroreport 12, 169–174. 10.1097/00001756-200101220-00041 11201080

[B18] Putkinen V. Tervaniemi M. Saarikivi K. de Vent N. Huotilainen M. (2014). Investigating the effects of musical training on functional brain development with a novel melodic MMN paradigm. Neurobiol. Learn. Mem. 110, 5–15. 10.1016/j.nlm.2014.01.007 24462719

[B19] Rüsseler J. Altenmüller E. Nager W. Kohlmetz C. Münte T. F. (2001). Event–related brain potentials to sound omissions differ in musicians and non–musicians. Neurosci. Lett. 308, 33–36. 10.1016/S0304-3940(01)01977-2 11445279

[B20] Schlaug G. Jäncke L. Huang Y. Staiger J. F. Steinmetz H. (1995). Increased corpus callosum size in musicians. Neuropsychologia 33, 1047–1055. 8524453 10.1016/0028-3932(95)00045-5

[B21] Schneider P. Scherg M. Dosch H. G. Specht H. Gutschalk A. Rupp A. (2002). Morphology of Heschl's gyrus reflects enhanced activation in the auditory cortex of musicians. Nat. Neurosci. 5, 688–694. 12068300 10.1038/nn871

[B22] Schröger E. Bendixen A. Denham S. L. Mill R. W. Bohm T. M. Winkler I. (2014). Predictive regularity representations in violation detection and auditory stream segregation: from conceptual to computational models. Brain Topogr. 27, 565–577. 10.1007/s10548-013-0334-6 24271978

[B23] Seppänen M. Brattico E. Tervaniemi M. (2007). Practice strategies of musicians modulate neural processing and the learning of sound-patterns. Neurobiol. Learn. Mem. 87, 236–247. 10.1016/j.nlm.2006.08.011 17046293

[B24] Seppänen M. Hämäläinen J. Pesonen A.-K. Tervaniemi M. (2012a). Music training enhances rapid plasticity of N1 and P2 source activation for unattended sounds. Front. Hum. Neurosci. 6:43. 10.3389/fnhum.2012.00043 22435057 PMC3303088

[B25] Seppänen M. Pesonen A.-K. Tervaniemi M. (2012b). Music training enhances the rapid plasticity of P3a/P3b event-related brain potentials for unattended and attended target sounds. Attent. Percept. Psychophys. 74, 600–612. 10.3758/s13414-011-0257-9 22222306

[B26] Shahin A. Bosnyak D. J. Trainor L. J. Roberts L. E. (2003). Enhancement of neuroplastic P2 and N1c auditory evoked potentials in musicians. J. Neurosci. 23, 5545–5552. 12843255 10.1523/JNEUROSCI.23-13-05545.2003 PMC6741225

[B27] Steele C. J. Bailey J. A. Zatorre R. J. Penhune V. B. (2013). Early musical training and whitematter plasticity in the corpus callosum: evidence for a sensitive period. J. Neurosci. 33, 1282–1290. 10.1523/JNEUROSCI.3578-12.2013 23325263 PMC6704889

[B28] Tervaniemi M. (2009). Musicians—same or different? Ann. N.Y. Acad. Sci. 1169, 151–156. 10.1111/j.1749-6632.2009.04591.x 19673771

[B29] Tervaniemi M. (2012). Musicianship—how and where in the brain?, in Musical Imaginations: Multidisciplinary Perspectives on Creativity, Performance and Perception, eds Hargreaves D. Miell D. MacDonald R. (Oxford: Oxford University Press), 285–295.

[B30] Tervaniemi M. Castaneda A. Knoll M. Uther M. (2006). Sound processing in amateur musicians and nonmusicians: event-related potential and behavioral indices. Neuroreport 17, 1225–1228. 10.1097/01.wnr.0000230510.55596.8b 16837859

[B31] Tervaniemi M. Huotilainen M. Brattico E. (2014). Melodic multi-feature paradigm reveals auditory profiles in music-sound encoding. Front. Hum. Neurosci. 8:496. 10.3389/fnhum.2014.00496 25071524 PMC4084670

[B32] Tervaniemi M. Rytkönen M. Schröger E. Ilmoniemi R. J. Näätänen R. (2001). Superior formation of cortical memory traces for melodic patterns in musicians. Learn. Mem. 8, 295–300. 10.1101/lm.39501 11584077 PMC311383

[B33] Virtala P. Huotilainen M. Partanen E. Tervaniemi M. (2014). Musicianship facilitates the processing of Western music chords—An ERP and behavioral study. Neuropsychologia 61, 247–258. 10.1016/j.neuropsychologia.2014.06.028 24992584

[B34] Vuust P. Brattico E. Glerean E. Seppänen M. Pakarinen S. Tervaniemi M.. (2011). New fast MMN paradigm for determining the neural prerequisites for musical ability. Cortex 47, 1091–1098. 10.1016/j.cortex.2011.04.026 21621766

[B35] Vuust P. Brattico E. Seppänen M. Näätänen R. Tervaniemi M. (2012). The sound of music: differentiating musicians using a fast, musical multi-feature mismatch negativity paradigm. Neuropsychologia 50, 1432–1443. 10.1016/j.neuropsychologia.2012.02.028 22414595

[B36] Vuust P. Pallesen K. J. Bailey C. van Zuijen T. L. Gjedde A.. (2005). To musicians, the message is in the meter. Neuroimage 24, 560–564. 10.1016/j.neuroimage.2004.08.039 15627598

